# Neural pathways associated with reduced rigidity during pallidal deep brain stimulation for Parkinson’s disease

**DOI:** 10.1152/jn.00155.2024

**Published:** 2024-08-07

**Authors:** Emily Lecy, Maria E. Linn-Evans, Sommer L. Amundsen-Huffmaster, Tara Palnitkar, Remi Patriat, Jae Woo Chung, Angela M. Noecker, Michael C. Park, Cameron C. McIntyre, Jerrold L. Vitek, Scott E. Cooper, Noam Harel, Matthew D. Johnson, Colum D. MacKinnon

**Affiliations:** ^1^Department of Neuroscience, University of Minnesota, Minneapolis, Minnesota, United States; ^2^Department of Neurology, University of Minnesota, Minneapolis, Minnesota, United States; ^3^Center for Magnetic Resonance Research, University of Minnesota, Minneapolis, Minnesota, United States; ^4^Department of Neurosurgery, University of Minnesota, Minneapolis, Minnesota, United States; ^5^Department of Biomedical Engineering, Duke University, Durham, North Carolina, United States; ^6^Department of Biomedical Engineering, University of Minnesota, Minneapolis, Minnesota, United States

**Keywords:** computational model, deep brain stimulation, globus pallidus, Parkinson’s disease, rigidity

## Abstract

Deep brain stimulation (DBS) of the internal segment of the globus pallidus (GPi) can markedly reduce muscle rigidity in people with Parkinson’s disease (PD); however, the mechanisms mediating this effect are poorly understood. Computational modeling of DBS provides a method to estimate the relative contributions of neural pathway activations to changes in outcomes. In this study, we generated subject-specific biophysical models of GPi DBS (derived from individual 7-T MRI), including pallidal efferent, putamenal efferent, and internal capsule pathways, to investigate how activation of neural pathways contributed to changes in forearm rigidity in PD. Ten individuals (17 arms) were tested off medication under four conditions: off stimulation, on clinically optimized stimulation, and on stimulation specifically targeting the dorsal GPi or ventral GPi. Quantitative measures of forearm rigidity, with and without a contralateral activation maneuver, were obtained with a robotic manipulandum. Clinically optimized GPi DBS settings significantly reduced forearm rigidity (*P* < 0.001), which aligned with GPi efferent fiber activation. The model demonstrated that GPi efferent axons could be activated at any location along the GPi dorsal-ventral axis. These results provide evidence that rigidity reduction produced by GPi DBS is mediated by preferential activation of GPi efferents to the thalamus, likely leading to a reduction in excitability of the muscle stretch reflex via overdriving pallidofugal output.

**NEW & NOTEWORTHY** Subject-specific computational models of pallidal deep brain stimulation, in conjunction with quantitative measures of forearm rigidity, were used to examine the neural pathways mediating stimulation-induced changes in rigidity in people with Parkinson’s disease. The model uniquely included internal, efferent and adjacent pathways of the basal ganglia. The results demonstrate that reductions in rigidity evoked by deep brain stimulation were principally mediated by the activation of globus pallidus internus efferent pathways.

## Graphical abstract

**Figure:**
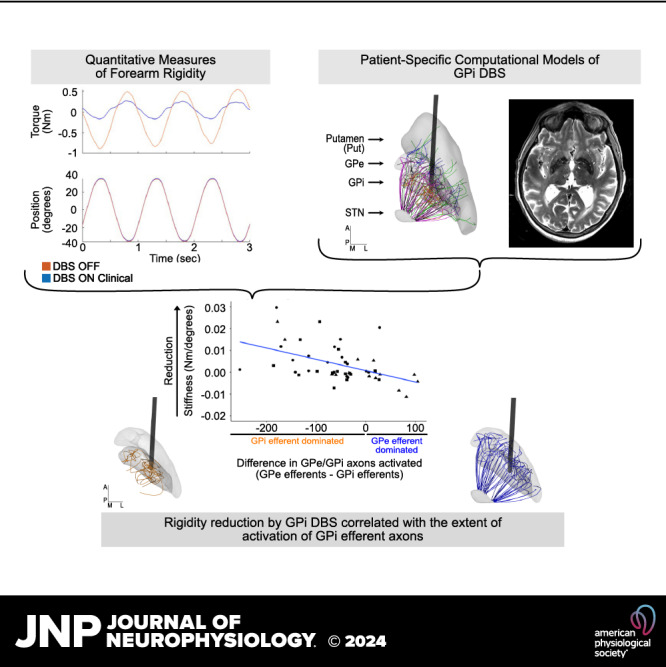

## INTRODUCTION

Muscular rigidity is one of the cardinal diagnostic motor signs leading to a diagnosis of Parkinson’s disease (PD) (1, 2) and is characterized by an abnormal increase in muscle activity in response to imposed muscle stretch, leading to enhanced resistance to passive joint motion. Rigidity is typically responsive to dopamine-replacement therapies (3, 4) and deep brain stimulation (DBS) of the subthalamic nucleus (STN) (4–6) or internal segment of the globus pallidus (GPi) (7–9), suggesting that degeneration of nigrostriatal dopaminergic pathways and disruption of basal ganglia function plays a critical role in the pathogenesis of this motor sign. In addition to its clinical importance, the assessment of rigidity is a valuable assay to interrogate the efficacy of DBS in both intraoperative and postoperative settings because it typically responds quickly to stimulation (within seconds to minutes) (10) and reductions in rigidity are often predictive of improvement in other motor signs (11). For this reason, clinicians often rely on the assessment of appendicular rigidity when programming DBS settings to determine the location (lead contact or contact combination) and stimulation parameters (current/voltage, pulse width, frequency) for optimal clinical effect (12).

Currently, there is no consensus on the optimal location to stimulate within GPi for treating parkinsonian rigidity. Early studies of the effects of posteroventral pallidotomy showed that a lesion in this region reduced rigidity by an average of 43% (13, 14), suggesting that disruption of output from the sensorimotor region of the GPi can be therapeutic. In keeping with this idea, Krack et al. (15) presented evidence that GPi DBS was most effective for reducing rigidity when the stimulation was delivered through contacts located in ventral regions of the posterior GPi. In contrast, other studies have shown that comparable improvements in rigidity could be induced by stimulation in central, dorsal, or ventral regions of the posterior GPi (16, 17). Retrospectively estimating the optimal stimulation location for suppressing rigidity based on clinical ratings of rigidity severity at clinically optimized settings is confounded by the fact that selection of the clinically optimal contact(s) and parameters is often heavily weighted by ordinal measures of improvements in bradykinesia, tremor, control of levodopa-induced dyskinesias, and other signs and by the use of ordinal scales to quantify symptoms (18, 19).

Computational models of GPi DBS (20, 21), supported by experimental studies (22, 23), have provided evidence that stimulation using common clinical parameters drives axonal activity in pathways that traverse the electric field, including the efferent output pathways of the stimulated structure and fibers of passage. Accordingly, the therapeutic effects of DBS are thought to be principally mediated by the modulation of abnormal neural spiking patterns into a more regular state, both within and downstream of the stimulated nucleus (24–26). In the context of GPi DBS, this suggests that stimulation through a contact located in the sensorimotor region likely activates pathways projecting to, and from, the GPi including efferent pathways to the thalamus and brain stem (via the lenticular fasciculus and ansa lenticularis) as well as axons of the direct (putamen to GPi) and indirect [external segment of the globus pallidus (GPe) to GPi and STN] pathways and other afferents (e.g., STN to GPe and GPi). The contributions of these pathways to the therapeutic effects of GPi DBS on rigidity are unknown.

Subject-specific computational models of axonal pathway activation, derived from anatomical and diffusion MRI, provide a tool to estimate the relative activations of pathways in the region of the stimulated contact (27–29). This approach has been used in the nonhuman primate model of parkinsonism to investigate the pathways contributing to the effects of GPi DBS on bradykinesia and rigidity (21). While multiple neuronal pathways were activated by DBS in the sensorimotor region of the GPi, improvements in clinical assessments of rigidity were observed with contacts located in both ventral and dorsal regions of GPi and ventral GPe. Model predictions showed that reductions in rigidity were associated with both pallidal efferent activation and current spread to the internal capsule such that direct activation of a small percentage of corticospinal axons contributed to reduced muscle tone (21).

In the present study, we translated the modeling pipeline developed by Johnson et al. (21) in nonhuman primates to study the pathways activated by GPi DBS in humans. The model was further updated with the addition of internal pathways of the basal ganglia in the region of the GPi and GPe including pallidothalamic, pallidosubthalamic, and striatofugal fibers. Subject-specific models were derived from reconstruction of the nuclei and axonal pathways [from preoperative ultrahigh-field 7-T MRI and postoperative computed tomography (CT)] (30, 31) in the region of interest in concert with finite element models (FEMs) and biophysical multicompartment neuron models. The outputs of the model were estimates of the percentage of axons exhibiting stimulation-induced action potentials in each pathway. We hypothesized that the level of reduction in forearm rigidity would be related to the percent activation of GPi efferents. As a secondary experiment, we compared the effects of stimulation directed at the dorsal versus ventral regions of the GPi on pathway activations and rigidity.

## METHODS

### Participants

Ten individuals (3 women, 7 men, age: 63.1 ± 8.2 yr; *n* = 17 GPi DBS leads) with Parkinson’s disease participated in this study (demographic and clinical details are provided in Table 1). Inclusion criteria were a diagnosis of idiopathic Parkinson’s disease by a movement disorders neurologist, an existing deep brain stimulation system implanted in the globus pallidus, and both presurgical 7-T MRI and postsurgical computed tomography (CT) scans. Individuals were excluded from the study if they had a history of musculoskeletal disorders affecting movements of the upper limbs, neurological disorders besides PD, history of dementia or cognitive impairment (Montreal Cognitive Assessment score, MoCA < 23), or postoperative complications that could affect participant safety or confound experimental results. All study procedures were approved by the University of Minnesota Institutional Review Board, and all participants provided written informed consent according to the Declaration of Helsinki.

**Table 1.** Demographics of study participants

ID	Sex	Age. yr	Years Since Diagnosis (at Time of Consent)	MDS-UPDRS III OFF	LDE, mg	Implant Side	DBS Device
*1*	M	53	15	56	696	Bilateral	Medtronic Legacy
*2*	M	56	11	43	1,010	Bilateral	Medtronic Legacy
*3*	F	82	11	50	825	Right	Abbott Infinity
*4*	M	71	8	48	950	Left	Medtronic Legacy
*5*	F	63	5	66	800	Bilateral	Abbott Infinity
*6*	F	60	9	51	1,000	Right	Abbott Infinity
*7*	M	62	11	67	150	Bilateral	Abbott Infinity
*8*	M	66	6	49	320	Bilateral	Abbott Infinity
*9*	M	64	9	67	800	Bilateral	Medtronic Legacy
*10*	M	54	5	52	850	Bilateral	Medtronic Legacy

Movement Disorder Society Unified Parkinson’s Disease Rating Scale III (MDS-UPDRS III) was performed OFF medication and OFF deep brain stimulation (DBS). Levodopa daily equivalent (LDE) was determined based on the Tomlinson formula.

### Imaging

Before DBS surgery, all participants underwent a 7-T MRI at the Center for Magnetic Resonance Research at the University of Minnesota using a Siemens console (Magnetom 7 T; Siemens, Erlangen, Germany), SC72 gradient coil, and 32-channel head coil (Nova Medical, Inc, Burlington, MA). The imaging protocol included high-resolution anatomical scans (T1 and T2 weighted) as well as diffusion-weighted imaging (DWI) using protocols described in previous publications (32, 33). One month after surgery, participants underwent a clinical CT scan with 0.6-mm cuts, which was registered with the T1-weighted MRI to allow for lead localization (30). Preprocessing corrections were performed to address image distortions inherent in this imaging pipeline. For the T1-weighted anatomical images, this included corrections for nonuniformity with FSL FAST (34), brain extraction with FSL BET (35), and coregistrations with T2-weighted images using FSL FLIRT with 6 degrees of freedom followed by a 12-degrees of freedom registration to make fine adjustments (36). For the DWI, FSL eddy and TOPUP were used to correct eddy current and susceptibility-induced distortions (37). Additionally, anatomical and DWI images were coregistered. Manual segmentations of basal ganglia nuclei, GPe, GPi, STN, putamen, and substantia nigra (SN), were extracted from the T2-weighted anatomical images by research staff experienced in this technique (30, 31, 33).

### Experimental GPi DBS Stimulation Settings

In addition to investigating the pathways activated by clinically optimized GPi DBS settings, this study sought to compare the effects of lower-amplitude dorsal and ventral GPi DBS on neural pathway activation and rigidity, which required the development of experimental DBS settings for each participant. StimVision, a software tool used to estimate the volume of tissue activated (VTA) by DBS, was used to identify stimulation settings that theoretically biased DBS to either dorsal or ventral regions of the GPi (38).

For each subject, the 7-T MRI data were used to create an anatomical model of each brain hemisphere (30). The manually segmented basal ganglia nuclei (GPe, GPi, STN, SN) were constructed into three-dimensional (3-D) volumes for visualization with the MRI slice data. The T1-weighted MRI was used as the base image for data coregistration, and the patient anatomical model was merged with the postoperative CT image to identify the position and orientation of each electrode in the brain. The subject-specific imaging data, anatomical volumes, and DBS electrode location were loaded into the StimVision software tool to assist with the definition of experimental DBS settings that would be used in the experimental sessions. Volume of tissue activated estimates of stimulation spread guided the selection of the contact and amplitude for the model-based DBS setting (39). The pulse width (60 µs) and stimulation frequency (130 Hz) were held constant for all DBS settings.

For each brain hemisphere, an expert in DBS modeling, blinded to the clinical settings (A.M.N. or C.C.M.), used the patient-specific StimVision model to select an electrode contact that most closely fit the anatomical designation of dorsal or ventral placement within the GPi. Next, VTA estimates were used to define a stimulation amplitude through the selected electrode contact that fulfilled the following criteria: *1*) concentrated VTA in the targeted anatomical area, *2*) avoided stimulation spillover to the nontargeted anatomical area (e.g., GPe with dorsal stimulation), and *3*) avoided stimulation spillover to the internal capsule. Definition of the StimVision experimental DBS settings was blinded to any information about the subjects, aside from the imaging data used to create the subject-specific DBS model. Experimental dorsal and ventral GPi and clinically optimized GPi DBS settings are presented in Table 2.

**Table 2.** Tested DBS settings

ID	Lead	Implant Side	Clinical Settings	Ventral Settings	Dorsal Settings
*1*	Medtronic Legacy	Left	1−2 − C +5 V, 130 Hz, 60 μs	1 − C +3 V, 120 Hz, 60 μs	3 − C +3 V, 120 Hz, 60 μs
		Right	1−2 − C +4.5 V, 125 Hz, 60 μs	1 − C +3 V, 120 Hz, 60 μs	3 − C +3 V, 120 Hz, 60 μs
*2*	Medtronic Legacy	Left	2 − C +3.6 V, 180 Hz, 60 μs	2 − C +2 V, 120 Hz, 60 μs	3 − C +2 V, 120 Hz, 60 μs
		Right	2 − C +3.5 V, 180 Hz, 60 μs	2 − C +2 V, 120 Hz, 60 μs	3 − C +2 V, 120 Hz, 60 μs
*3*	Abbott Infinity	Right	2(abc) − C +4 mA, 130 Hz, 90 μs	2(bc) − C +3 mA, 120 Hz, 60 μs	4 − C +3 mA, 120 Hz, 60 μs
*4*	Medtronic Legacy	Left	2 −C +3.5 V, 130 Hz, 60 μs	1 − C +2 V, 120 Hz, 60 μs	2 − C +2 V, 120 Hz, 60 μs
*5*	Abbott Infinity	Left	3(c) − C +3 mA, 200 Hz, 90 μs	2(c) − C +1 mA, 120 Hz, 60 μs	4 − C +2 mA, 120 Hz, 60 μs
		Right	3(abc) − C +4 mA, 200 Hz, 90 μs	2(b) − C +1 mA, 120 Hz, 60 μs	4 − C +2 mA, 120 Hz, 60 μs
*6*	Abbott Infinity	Right	2(abc) − C +3.8 mA, 130 Hz, 60 μs	2(ab) − C +2.5 mA, 120 Hz, 60 μs	4 − C +2.5 mA, 120 Hz, 60 μs
*7*	Abbott Infinity	Left	3(abc) − C +3.7 mA, 130 Hz, 90 μs	2(b) − C +2 mA, 120 Hz, 60 μs	3(b) − C +2 mA, 120 Hz, 60 μs
		Right	2(abc) − C+3.7 mA, 130 Hz, 90 μs	2(a) − C +2 mA, 120 Hz, 60 μs	3(a) − C +2 mA, 120 Hz, 60 μs
*8*	Abbott Infinity	Left	2(abc) − C +4 mA, 130 Hz, 60 μs	3(bc) − C +4 mA, 120 Hz, 60 μs	4 − C +4 mA, 120 Hz, 60 μs
		Right	2(abc) − C +4 mA, 130 Hz, 60 μs	2a(c) − C +4 mA, 120 Hz, 60 μs	3 − C +4 mA, 120 Hz, 60 μs
*9*	Medtronic Legacy	Left	2–3 +3.7 V, 130 Hz, 90 μs	2 − C +2 V, 120 Hz, 60 μs	3 − C +2 V, 120 Hz, 60 μs
		Right	2(abc) − C +3.7 mA, 130 Hz, 90 μs	1 − C +2 V, 120 Hz, 60 μs	3 − C +2 V, 120 Hz, 60 μs
*10*	Medtronic Legacy	Left	2 − C+3.4 V, 130 Hz, 60 μs	0 − C +2 V, 120 Hz, 60 μs	2 − C +2 V, 120 Hz, 60 μs
		Right	1 − C+2.8 V, 130 Hz, 60 μs	1 − C +2 V, 120 Hz, 60 μs	3 − C +2 V, 120 Hz, 60 μs

The active contacts are listed under the clinical, dorsal, and ventral settings columns. The cathode is indicated as a number followed by a – (e.g., 3 −, indicating that contact 3 is set as the cathode). The anode is indicated with a + sign. In instances where the case (implanted pulse generator) is set as the anode, this is abbreviated with a C (e.g., C +). For settings involving segmented electrode contacts, the active segments are indicated in parentheses following the contact number. Below the contact information, amplitude, frequency, and pulse width are listed. DBS, deep brain stimulation.

### Pallidal DBS Pathway Activation Model

**Figure 1.**
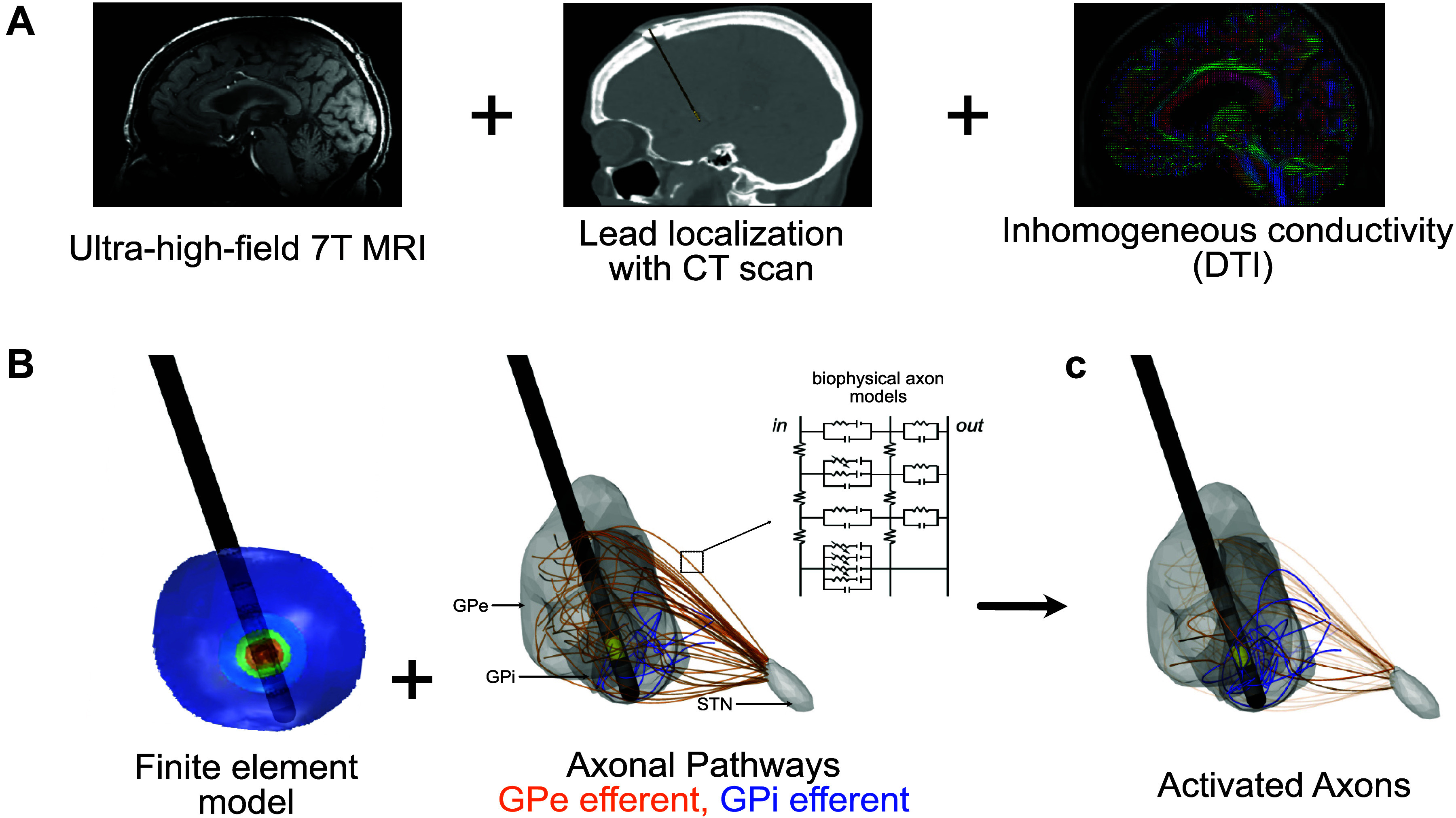
Subject-specific internal segment of the globus pallidus (GPi) deep brain stimulation (DBS) computational modeling pipeline. *A*: finite element models were data-driven from preoperative MRI and postoperative computed tomography (CT) scans. *B*: the combination of the finite element model and biophysical axon models enabled determination of the stimulation threshold for eliciting action potentials within each axon within a pathway. *C*: model-predicted axonal activation is shown in orange [external segment of globus pallidus (GPe) axons passing through the GPi to the subthalamic nucleus (STN) (GPe-GPi-STN)] and blue (GPi efferents).

Subject-specific computational models were constructed for all participants by using models of stimulation-induced tissue potentials coupled with biophysical multicompartment neuron models to estimate the extent of activation of neural pathways in the vicinity of the stimulating electrode(s) (Fig. 1).

#### Pallidal populations.

**Figure 2.**
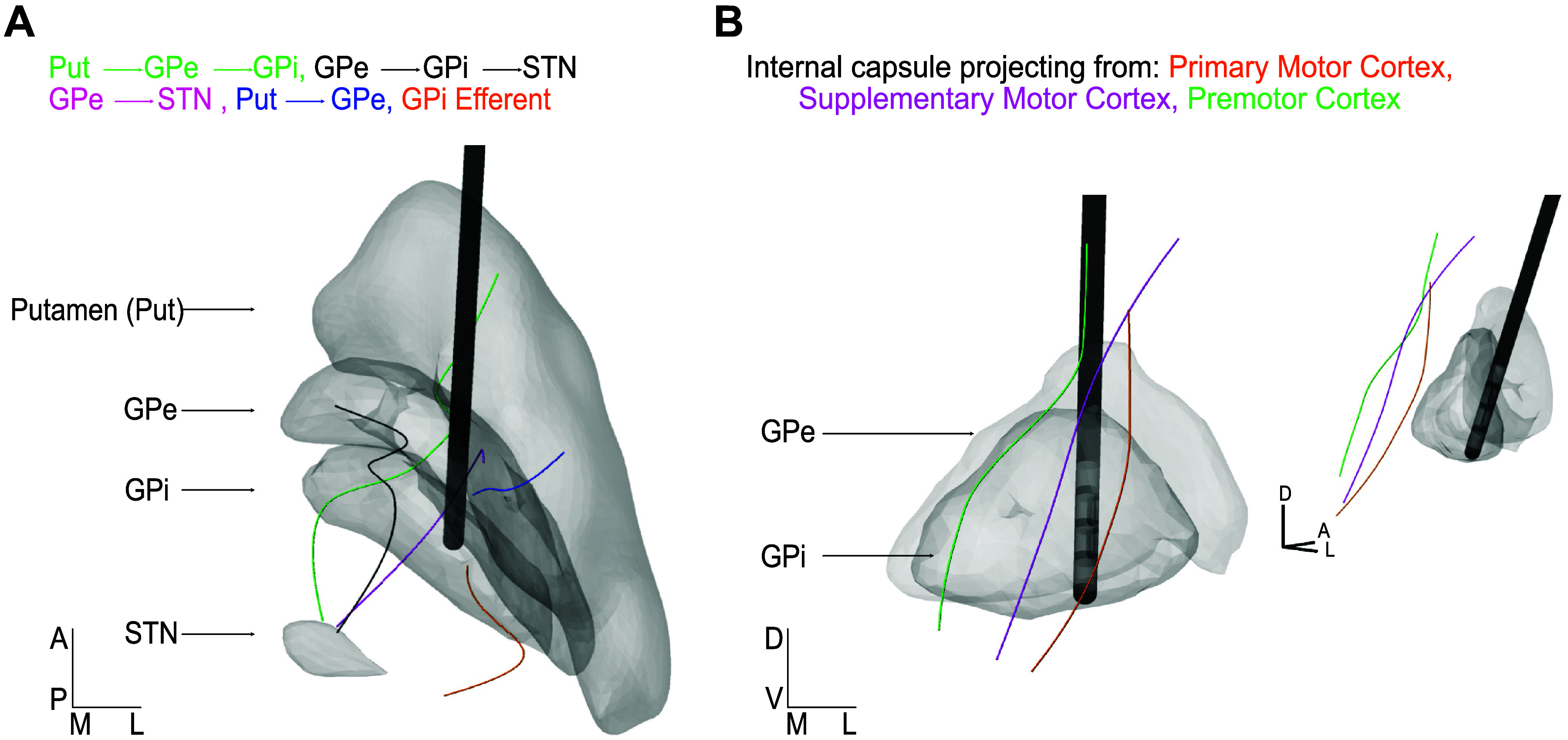
Reconstructions of axons passing through or adjacent to the internal segment of the globus pallidus (GPi). *A*: axons traversing through both external (GPe) and internal (GPi) segments of the globus pallidus. Axons were reconstructed with curvatures based on nonhuman primate single-axon histological reconstructions (40–43). *B*: corticospinal tract axon examples from 2 viewpoints, representative of 3 subtracts, that project from motor, premotor, and supplementary motor area cortical regions. Images are oriented anatomically: anterior (A), posterior (P), medial (M), lateral (L), dorsal (D), and ventral (V). STN, subthalamic nucleus.

Projections through the GP were constructed in MATLAB and populated within the segmented nuclei of interest. Waypoints within each nuclei were connected via a makima interpolation fitting algorithm to form axonal fibers (Fig. 2*A*).

##### GPi efferent pathways.

GPi efferents originated at randomly populated points within the GPi. Upon exiting the nucleus, axons were connected to either a dorsal or a ventral waypoint contour (50:50) (44).

##### Pallidosubthalamic pathways.

Two pallidosubthalamic pathways were modeled: *1*) GPe axons passing through the GPi to the STN (GPe-GPi-STN) and *2*) direct GPe projections to STN (GPe-STN). The percentage of GPe projections passing through GPi (37.5%) versus traveling directly to STN (62.5%) was determined from nonhuman primate histological studies (40–43).

##### Striatofugal pathways.

Two striatofugal pathways were modeled: *1*) projections from the putamen to GPe (Put-GPe) and *2*) projections from the putamen that passed through the GPe to the GPi (Put-GPe-GPi). The percentage of putamen projections that ended in the GPe (49%) versus those that traversed through the GPe and GPi (51%) were determined based on primate histological studies (40, 43).

**Figure 3.**
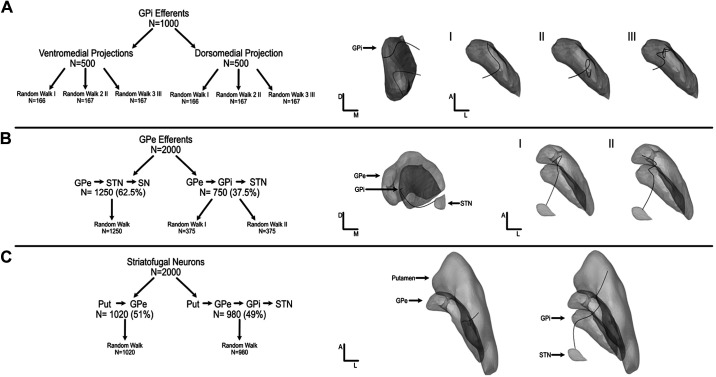
Axon breakdown for all internal pathways showing different types of axon curvature that could be randomly created in each pathway for internal segment of the globus pallidus (GPi) efferents (*A*), external segment of the globus pallidus (GPe) efferents (*B*), and striatofugal fibers (*C*). All fiber curvature is based on nonhuman primate single-axon tracing studies (40–43). Put, putamen; SN, substantia nigra; STN, subthalamic nucleus.

All fibers of passage had a different number of waypoints, creating different levels of movement and turns that were constrained by trajectories observed in nonhuman primate axon tracing studies to ensure that axons were not exhibiting abnormally sharp turns and movement (43, 45, 46) (Fig. 3). Full-scale models contained 250–1,250 axons per pathway (Table 3).

**Table 3.** Neural pathway populations for GPi DBS computational model

Pathway	Total Axons	Average Removed (±SD)
GPi efferent fibers	1,000	16.2 ± 8.7%
GPe → GPi → STN	750	11.6 ± 3.6%
GPe → STN	1,250	6.7 ± 1.6%
Putamen → GPe → GPi	980	13.7 ± 5.8%
Putamen → GPe	1,020	4.8 ± 2.7%
Internal capsule projecting to primary motor cortex	500	0.42 ± 1.7%
Internal capsule projecting to premotor cortex	250	0.05 ± 0.19%
Internal capsule projecting to supplementary motor cortex	250	0.24 ± 0.97%

For each pathway, the total number of axons populated was determined through primate histological studies (40–43). Axons that came in contact with the encapsulation layer around the lead were removed from subsequent analysis. The average number of axons removed from each pathway across the deep brain stimulation (DBS) leads modeled in this study is shown with SD. GPe, external segment of the globus pallidus; GPi, internal segment of the globus pallidus; STN, subthalamic nucleus.

#### Internal capsule.

Three capsular pathways were constructed to project from primary motor cortex (M1, Brodmann’s area 4), premotor cortex (PM, part of Brodmann’s area 6), and supplementary motor cortex (SMA, part of Brodmann’s area 6). Pathways were reconstructed using probabilistic tractography in FSL Probtrackx (47). Fiber orientations were estimated at each voxel using FSL bedpostx with a maximum of 3 crossing fibers. The seed masks for the cortical areas of M1 and BA6 were acquired with the Freesurfer image analysis suite [BA6 was further divided into PM and SMA based on individual atlas alignment (44), and a manually segmented cerebral peduncle mask was used as the waypoint (48)]. Full-scale models showed overlap among these three tracts on the anterior-posterior axis (44) but did have an anterior to posterior order as shown in Fig. 2*B*.

#### Multicompartment axon models.

Each of the resulting fiber tracts was populated with multicompartment cable models of myelinated axons with a fiber diameter of 2 µm for pallidal populations (49) and 5.7 µm for internal capsule axons (27). The multicompartment axon models consisted of nodes of Ranvier, myelin attachment segments, paranode main segments, and internode segments with membrane dynamics consistent with those used in previous studies (20, 21, 28). Pathways were populated to ensure that all aspects of the respective nuclei were sufficiently covered with axons. Axons that intersected with the lead or a 0.25-mm-thick encapsulation layer around the lead were removed from the model to account for axonal damage during lead insertion (Table 3).

#### Finite element model.

Each participant’s DBS lead(s) and imaging-derived brain anatomy were incorporated into a subject-specific finite element model (FEM) using COMSOL Multiphysics 5.4. To account for varying conductivities within the brain, FSL’s FAST tool was used to segment the T1-weighted anatomical images into white matter, gray matter, and cerebrospinal fluid (36, 50). Conductivity tensors were then estimated for each voxel of the participant’s DWI and imported into COMSOL to account for inhomogeneity and anisotropy within the brain in the FEM (51, 52). The conductivity tensors were calculated based on scaling the diffusion matrix at each voxel by the isotropic conductivities of the corresponding tissue (0.11 S/m for gray matter and 0.065 S/m for white matter) (53). Other tissues were modeled with isotropic electrical properties (0.3 S/m). Brain surface geometry extracted from the T1-weighted image with FSL BET was smoothed with 3D Slicer’s Gaussian smoothing tool and imported as a surface into the model. The DBS lead (Medtronic 3389, Medtronic 3387, or Abbott Infinity 6172), a 0.25-mm-thick isotropic (0.3 S/m) encapsulation layer surrounding the lead, and a generic cranium profile were also included in the FEM. For current-controlled systems, a normal current density was applied to the active electrode surfaces by dividing a 1-mA pulse amplitude by the surface area of the electrode. For voltage-controlled systems, a 1-V electric potential was applied to the active electrode. The base of the neck was set as ground, while the rest of the head surface and lead shaft had a current density of zero. Simulations were run at a single AC frequency of 3,049 Hz or 4,294 Hz based on the median frequency of the waveform used in clinical DBS for either a current- or a voltage-controlled device. The finite element analysis was solved using COMSOL’s AC/DC module. The extracellular voltages predicted by the FEM were interpolated at each compartment of the multicompartment cable axon models.

#### Simulating axonal pathway activation.

Stimulation of the modeled axons was performed with NEURON, a programming environment designed for solving differential equations underlying the dynamics of neuronal membrane biophysics (54). For each participant, a 10-pulse current-controlled or voltage-controlled waveform was applied using the pulse width and frequency of the subject-specific DBS settings (Table 2). The waveforms were filtered to reflect filtering that occurs to electrical stimulus pulses passing through neural tissue (55). This waveform was then applied to each compartment of the axonal model, and axons were considered “activated” if action potentials were elicited within 10 ms after at least 80% of the 10 simulated stimulus pulses. A binary search algorithm was then employed to determine the stimulus amplitude threshold (±0.1 mA or V) required to activate each axon for each stimulation setting. The ratio of activated to nonmodulated axons was used to define percent activation for each pathway.

### Experimental Protocol and Quantitative Rigidity Measures

**Figure 4.**
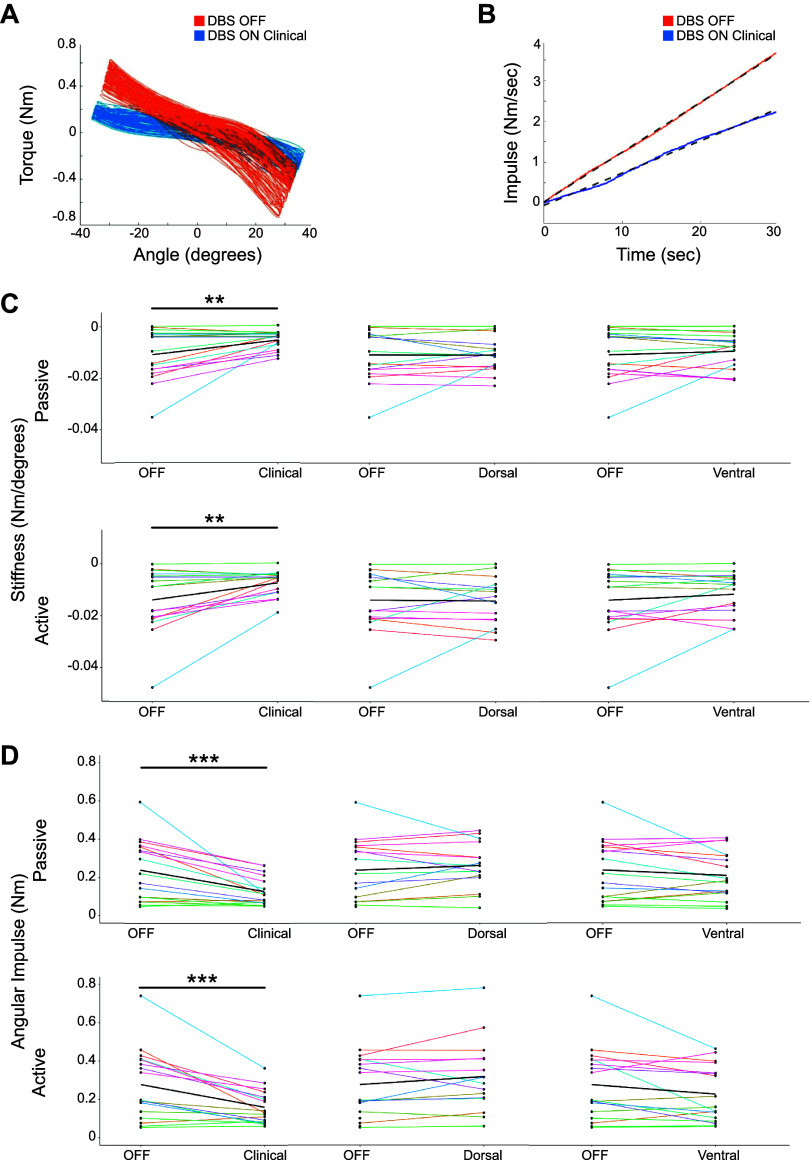
Change in rigidity measures with internal segment of the globus pallidus (GPi) deep brain stimulation (DBS). *A*: trace of position and torque taken from an upper extremity robotic manipulandum in both DBS OFF and DBS ON settings from a representative participant. *B*: traces of impulse (integrated torque) in both DBS OFF and DBS ON settings from a representative participant. The dashed line shows the line of best fit for the impulse, the slope of which is defined as angular impulse. *C* and *D*: changes in stiffness (*C*) and angular impulse (*D*) across DBS settings for all participants. *Top*: passive measures. *Bottom*: trials in which an activation maneuver was completed. The thick black line indicates the average change in rigidity scores between OFF and the tested setting, and thin colored lines are individual responses. Paired Wilcoxon signed-rank tests compared changes in rigidity differences between OFF and ON-DBS settings. **P ≤ 0.005, ***P ≤ 0.0005.

Participants completed separate blinded and randomized data collection visits for each DBS setting tested (OFF, clinical, dorsal, ventral). Participants were tested in the practically defined OFF medication state (after overnight, 12+ h from regular-release Parkinson’s medications, and 24 h from any extended-release Parkinson’s medications). Visits were separated by at least 1 wk. On the day of data collection, DBS parameters were set by a movement disorders neurologist not involved in data collection to ensure that participants and study staff were blinded to the setting. All testing occurred after a 1-h DBS wash-in period, after which motor symptom severity was assessed with the Movement Disorder Society Unified Parkinson’s Disease Rating Scale III (MDS-UPDRS Part III) by a certified and experienced rater. Quantitative measures of forearm rigidity on the side contralateral to DBS (right and left arm tested separately in participants with bilateral GPi DBS) were obtained with a custom-built robotic manipulandum (56). This device imposed a sinusoidal angular trajectory about the supination-pronation axis of ±40° at 1 Hz while collecting measures of the resistive torque. The position of the forearm and angle of the elbow and shoulder were standardized within and between sessions. During rigidity testing, the robotic manipulandum passively moved the participant’s forearm contralateral to the GPi DBS lead implant, while the ipsilateral forearm was either at rest (passive condition; forearm resting on their lap) or actively performing a tapping task (active condition; tapping their hand on their lap as “big and fast as possible”). Examples of each quantitative output are shown in Fig. 4, *A* and *B*. Each trial lasted 45 s, resulting in the collection of a minimum of 30 oscillation cycles. The primary outcome measures [determined with MATLAB (MathWorks, Natick, MA)] were stiffness (the slope of torque vs. angular displacement) and the angular impulse (calculated from a 1st-order regression line fit to the rectified torque data vs. time; the slope of that line was used as the measure of angular impulse). Both stiffness and angular impulse are variables used previously and correlate well to clinical ratings (56–58). Additionally, quantitative measures of rigidity derived from the manipulandum have been shown to significantly correlate with UPDRS rigidity scores (56).

### Statistics

Statistical analyses were computed in RStudio. The impact of DBS on rigidity scores was examined with paired Wilcoxon signed-rank tests comparing rigidity scores with each DBS setting versus OFF scores.

Linear mixed effects models (LMEs) were used to examine the relationship between pathway activations and rigidity outcomes. LMEs were created using the lmer package and followed the base equation of primary rigidity output ∼ GPi Efferent + GPeGPiSTN + GPeSTN + PutGPe + PutGPeGPi + (1|Hemisphere). Fixed effects are the raw number of axons per pathway activated by DBS. Hemisphere was treated as a random intercept, as each hemisphere has varying lead placement and programmed settings. Models were created based on the number of axons activated to account for differences in percentage activations that may arise from uneven deletion of fibers that overlapped with the lead or the lead encapsulation (Table 3). Internal capsule pathways did not show activation in 85% of the stimulation conditions (43/51). A principal component analysis further showed that all capsular pathways lacked variability in pathway activation; thus they were not included in statistical analysis. Outputs of the LMEs were used to create a dataset of predicted change in rigidity scores based on the number of activated axons in each pathway, allowing us to see the accuracy of the model. Model-predicted values were compared to the actual measured values for each subject, and a Kendall’s rank correlation was computed to evaluate the predictive value of each model.

An exploratory analysis using the relative differences of activation in GP efferents was used to further investigate the relationship between pathway activation and the changes in rigidity. Differences were calculated between the number of GPe and GPi efferents (GPe efferents − GPi efferents). LMEs were again used, with hemisphere as a random effect. Individual pathways (GPi efferent or GPe efferents) were run through models separately, as individual pathway analysis showed the lowest Bayesian information criterion and therefore best model fit.

Secondary analyses of pathway activation based on stimulation location (clinical, dorsal, and ventral settings) were also completed. Distance measures were derived by the dorsal-ventral distance from the center of the stimulating electrode(s) to the most ventral point in the GPi. This distance measure was put into a general linear model versus pathway activation to assess the impact of location of percent activation. The effects of pathway activation on dorsal versus ventral stimulation were also analyzed by paired Wilcoxon signed-rank tests between the percent activation of dorsal and ventral settings for each modeled pathway.

## RESULTS

### Effects of GPi DBS on Rigidity

Clinically optimized GPi DBS resulted in a significant reduction in forearm rigidity measures for both the passive (stiffness: *P* = 0.0082; angular impulse: *P* < 0.001) and contralateral activation (stiffness: *P* = 0.0041; angular impulse: *P* < 0.001) conditions compared to OFF stimulation (paired Wilcoxon signed-rank tests). There were no significant differences in rigidity measures between the OFF stimulation and either the dorsal or ventral settings in passive (stiffness: *P* = 0.84, *P* = 0.95; angular impulse: *P* = 0.72, *P* = 0.26) and active (stiffness: *P* = 0.64, *P* = 0.46; angular impulse: *P* = 0.14, *P* = 0.08) (paired Wilcoxon signed-rank test) conditions. The absence of an effect of dorsal or ventral stimulation was likely due to the relatively low stimulation amplitudes compared with the clinically optimized DBS settings (Fig. 4).

### Pathway Activation Differs across Stimulation Location

**Figure 5.**
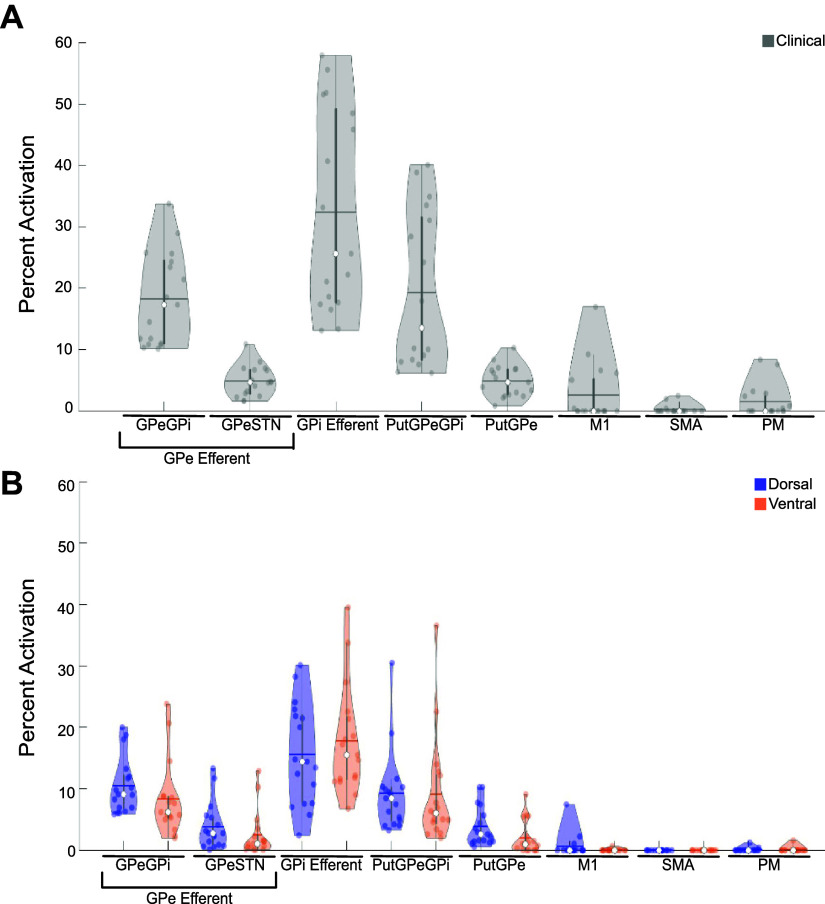
Violin plots showing pathway activation distributions across clinically optimized stimulation (*A*) and low-amplitude dorsal and ventral settings (*B*) in all participants. Dots are individual data points (*N* = 17). Solid colored horizontal lines represent means; open circles represent the median. All colored dots are individual data points. GPe, external segment of the globus pallidus; GPi, internal segment of the globus pallidus; M1, primary motor cortex; PM, premotor cortex; Put, putamen; SMA, supplementary motor cortex; STN, subthalamic nucleus.

**Figure 6.**
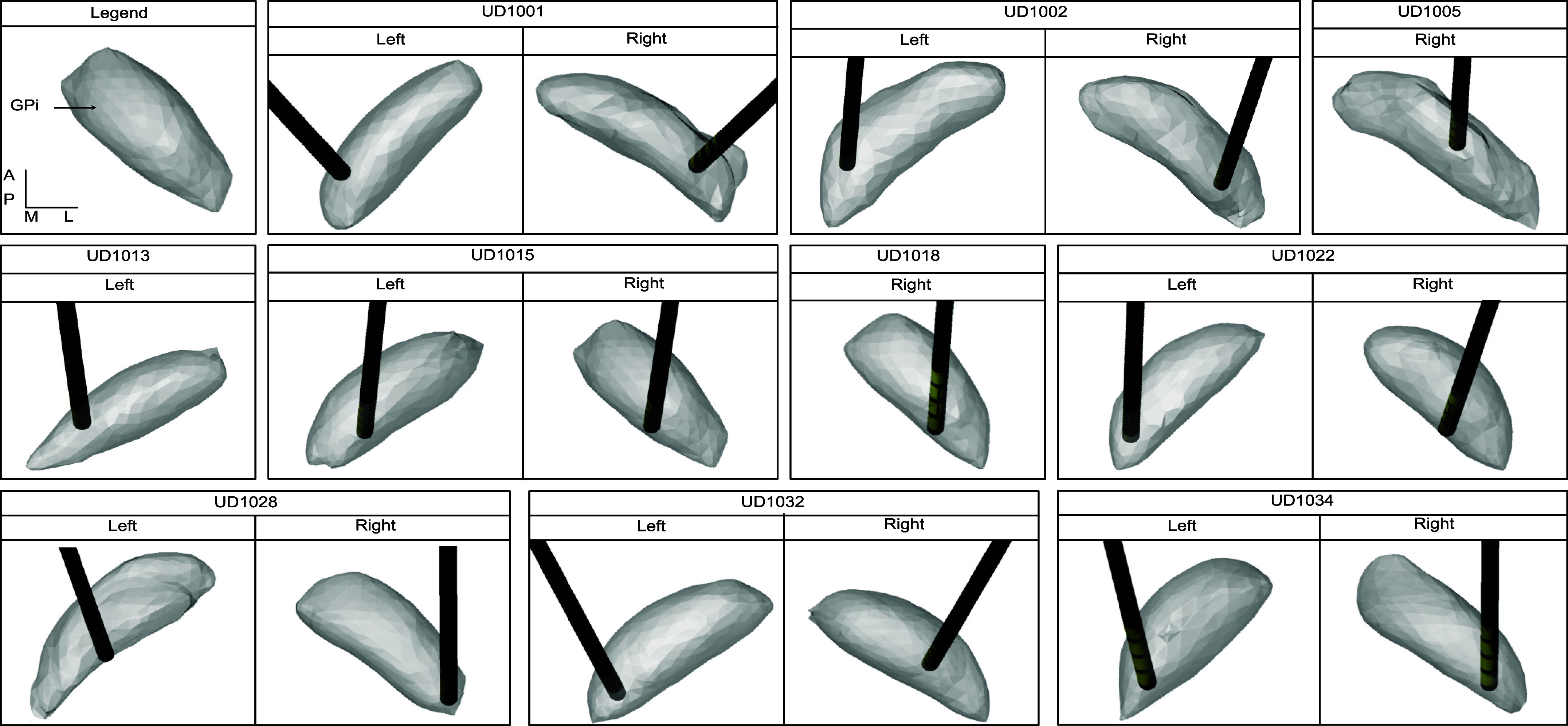
Electrode localization within the internal segment of the globus pallidus (GPi) for all leads. Viewpoints are from superior to the GPi, with direction labeled in the key. Images are oriented anatomically: anterior (A), posterior (P), medial (M), lateral (L), dorsal (D), ventral (V).

Stimulation using clinically optimized settings typically resulted in activation of all modeled basal ganglia pathways. The highest percentage activations were observed in the GPi efferent, GPe-GPi, and putamen-GPe-GPi pathways (Fig. 5*A*). Internal capsule activations, particularly M1 projections, were observed in only a subset of participants and usually were relatively low in comparison to basal ganglia pathway activations. The relatively low activation of the internal capsule was likely due to the consistently lateral and dorsal placement of the clinical contact relative to the ventromedial border of the GPi (Fig. 6).

Pathway activation for the dorsal and ventral model settings was lower than for the clinical setting principally because of the use of lower stimulation amplitudes (Fig. 5*B*). Stimulation through dorsal contacts was associated with significantly higher activation of GPe-STN and Put-GPe pathways compared to ventral contacts (*P* = 0.010, *P* = 0.0029). There was no significant difference (*P* = 0.45) in GPi efferent activation between stimulation through ventral and dorsal contacts.

**Figure 7.**
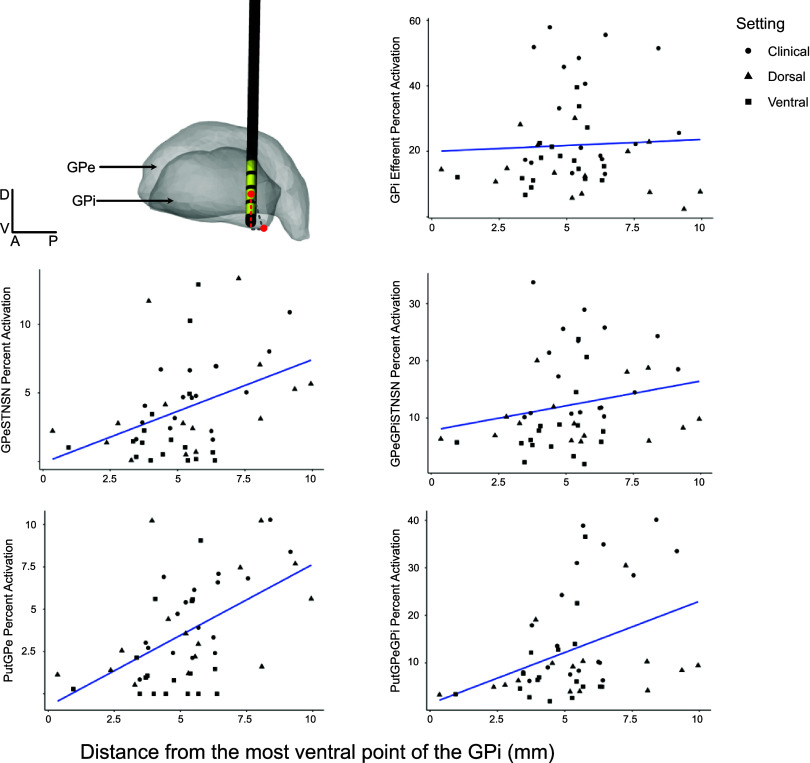
Relationship between the distance from the most ventral point of the internal segment of the globus pallidus (GPi) and neural pathway activation of all internal fibers of passage. *Top left* quadrant shows how distance was calculated, using an example participant: by taking the distance along the dorsal-ventral plane (dashed red line) between the most ventral point of the GPi and the center of the active electrode. Five scatterplots show the relationship between the distance from the ventral GPi and pathway activation across internal axonal pathways. Trend lines were created using a general linear model of pathway activation by distance. Stimulation settings are shown in shapes according to the key. GPe, external segment of the globus pallidus; Put, putamen; STN, subthalamic nucleus; SN, substantia nigra.

Although there was no significant relationship between GPi efferent activation and distance of the stimulation contact from the nearest ventral border of the GPi (Fig. 7; *P* = 0.72), the distance of the contact from the ventral GPi border had a significant correlation to the activation of fibers traversing from the putamen and branching in GPe and fibers projecting from the GPe and traversing around the GPi and to the STN (PutGPe, *P* = 0.000078, *R* = 0.53, GPeSTNSN, *P* = 0.0025, *R* = 0.42), with fibers being preferentially activated with dorsal locations (Fig. 7).

### Relationship of Pathway Activation to Upper Extremity Rigidity

**Figure 8.**
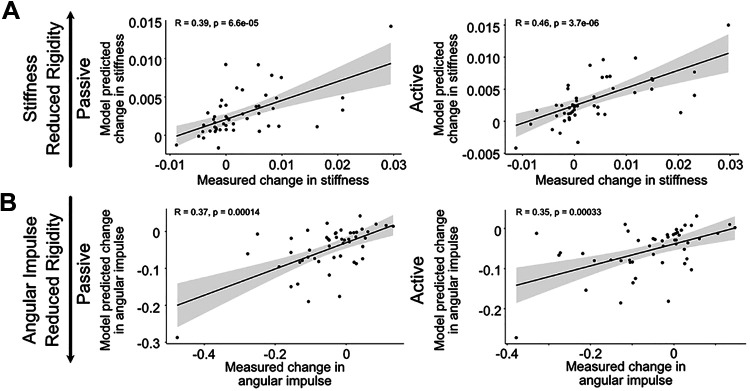
Model predicted change in rigidity scores (*y*-axes) compared to measured change in rigidity scores (*x*-axes) for stiffness (*A*) and angular impulse (*B*) in passive (*left*) and active (*right*) conditions. Linear mixed effects model (LME) intercept and pathway slopes were fed into an equation that used actual pathway activation to predict change in rigidity scores. The relationship between the measured change in rigidity scores and the LME predicted scores was assessed with a Kendall’s rank correlation coefficient (*R* and *P* values shown on each plot). The regression line is shown in blue, with the 95% confidence interval indicated by the gray shading.

Linear mixed effect models that included all modeled pathways were run to test whether any pathway had a significant contribution to the change in rigidity. GPi efferent axon activation was associated with a significant reduction in both passive and active conditions for angular impulse (passive: *P* = 0.0065; active: *P* = 0.034) and stiffness (passive: *P* = 0.045; active: *P* = 0.0092). PutGPe fiber activation was also associated with a significant reduction in stiffness (passive: *P* = 0.032, active: *P* = 0.037) and passive angular impulse [passive: *P* = 0.020, active: not significant (NS), *P* = 0.090]. In contrast, GPeSTN fiber activation was associated with increased stiffness and angular impulse, but these relationships did not reach threshold of significance (stiffness: passive *P* = 0.097, active *P* = 0.076; angular impulse: passive *P* = 0.059, active *P* = 0.12). The LMEs were used to create a dataset of predicted change in rigidity scores based on the percentage of activated axons in each pathway, allowing us to evaluate the predictive accuracy of the model. These predictive outcomes showed significant correlations between the model-predicted percent change in rigidity and the measured change in rigidity for stiffness (passive: *P* = 0.00007, active: *P* = 0.000004) and angular impulse (passive: *P* = 0.00014, active: *P* = 0.00033) as indicated by the results of the Kendall’s rank correlation (Fig. 8).

**Figure 9.**
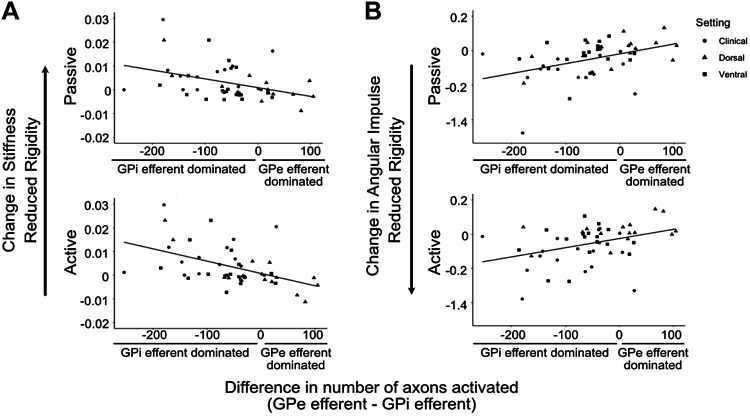
Scatterplots showing the relationship between stiffness (*A*) and angular impulse (*B*) and pathway activation differences in both passive (*top*) and active (*bottom*) conditions. The *x*-axis represents the difference in the number of axons activated between external segment of the globus pallidus globus pallidus (GPe) efferent (GPeSTNSN, GPeGPiSTNSN) and internal segment of the globus pallidus (GPi) efferent pathways by GPi deep brain stimulation (DBS) (GPe efferents − GPi efferents). Motor output measures are shown as changes, calculated by subtracting the measured outcome between DBS-ON and DBS-OFF conditions. Settings are represented in shapes according to the key. STN, subthalamic nucleus; SN, substantia nigra.

An exploratory analysis used LME models to investigate whether the relative differences of activation in GP efferents induced changes in rigidity (Fig. 9). Forearm stiffness and angular impulse were significantly related to preferential activation of GPi efferents (stiffness: passive *P* = 0.005, active *P* = 0.01; angular impulse: passive *P* < 0.001, active *P* = 0.005).

## DISCUSSION

This study is the first to investigate the pathways mediating changes in parkinsonian rigidity with human globus pallidus DBS using subject-specific computational models. The study translated a previous model, tailored to nonhuman primates (20, 21), to humans and incorporated a larger set of efferent projections, including those from the GPi, internal capsule (M1, SMA, PM), and internal pathways of the basal ganglia (putamen to GPe and GPi and GPe to STN and GPi). The computational model enabled comparisons between the relative activations of the striatofugal and GP efferent pathways across multiple DBS configurations and assessment of how those activations relate to changes in forearm rigidity. Quantitative measures of forearm rigidity were obtained with a robotic manipulandum, allowing for objective assessment of the effects of DBS. The data show that activation of GPi efferent and PutGPe pathways are associated with reductions in forearm rigidity in humans.

In keeping with the results of clinical trials of GPi DBS, stimulation using the clinically optimized contact(s) and parameters resulted in a significant reduction in clinical ratings of the severity of rigidity (7, 8, 59). Metrics of rigidity derived from the robotic manipulandum (56) also showed that forearm pronation/supination stiffness angular impulse were significantly reduced during passive resting movement of the limb, with and without a contralateral activation maneuver. On average, clinical stimulation reduced forearm stiffness by 35% during the passive condition. Similar findings have been reported for STN DBS, showing that stimulation in the off-medication state was associated with an ∼46% reduction in passive quantitative measures of elbow joint rigidity (work done) (4). Currently, the mechanisms by which STN DBS reduces rigidity, and whether the pathways mediating these effects are similar to GPi DBS, are unknown.

Previous studies that have examined the putative mechanisms contributing to reductions in rigidity with GPi DBS have predominantly focused on the relationships to stimulation contact location within the nucleus. Intraoperative studies of the acute effects of high-frequency stimulation showed that reductions in rigidity could be evoked with stimulation in central, ventral, and dorsal regions of the GPi, GPi-GPe border, and ventral GPe (16, 17). Similarly, studies in people with chronically implanted DBS have demonstrated that rigidity can be suppressed with stimulation irrespective of contact location across the ventral-dorsal axis of the GPi (60, 61), or even via ventral GPe stimulation (62). Retrospective analyses of the contact locations providing optimal clinical outcomes, which are heavily weighted by assessments of bradykinesia, rigidity, and tremor, have shown that there is considerable variability in the location of the effective contact across individuals (18, 19, 63). These findings demonstrate that stimulation location, and the volume of tissue activated by the induced electric field, are not the sole factor contributing to therapeutic effects.

The current understanding of DBS therapy focuses on an activation-based mechanism, whereby DBS drives axonal activity to modulate neural patterns into a more regular state across a functional network (24–26, 64). Axonal activation is dependent on not only the stimulation parameters (amplitude, frequency, pulse width) but the location, orientation, and curvature of the axon relative to the stimulating electrode (65, 66). Using subject-specific computational models, our study shows that GPi DBS is not selective to one axonal pathway and instead activates multiple pathways, including GPi efferents, GPe-related pathways, and putamenal efferent pathways, which then impact the function of the basal ganglia-thalamocortical network. Accordingly, changes in contact location and stimulation parameters affect the relative contributions of these pathways in basal ganglia output. We found no significant relationship between the location of the clinical contact relative to the dorsal border of the GPi and the relative activation of GPi output axons, demonstrating that basal ganglia output can be comparably driven by dorsal or ventral stimulation. However, it is important to note that our model did not incorporate topographically organized motor subcircuits of the GPi output (e.g., primary motor vs. supplementary motor area) (67) and differences in the relative contributions of these output pathways may play a critical role in clinical outcomes. In contrast, the ventro-dorsal location of the contact had a significant influence on the activation of internal pathways of the basal ganglia. Stimulation through more dorsal contacts was associated with higher relative activations of GPe-STN and Put-GPe pathways compared to more ventral contacts. A linear mixed effects model that incorporated the activations of all modeled pathways showed that reductions in forearm rigidity were associated with preferential activation of GPi efferents and PutGPe axons.

Previous studies have provided evidence that activation of the internal capsule may also contribute to the suppression of rigidity with DBS. Using subject-specific computational models of GPi DBS in nonhuman primates, Johnson et al. (21) showed that reductions in rigidity scores correlated with the spread of axonal activation into the internal capsule (motor-related axons), albeit at low-level activation levels, in conjunction with activation of GPi and GPe efferents. In the present study, activation of the corticospinal tract of the internal capsule was not uniformly observed across all participants; thus, there was not a clear association between axonal activation in any of the topological tracts modeled within internal capsule and changes in muscle rigidity. The relatively low levels of internal capsule activation likely stem from the surgical approach in this study of placing DBS leads in the posterolateral sensorimotor territory in GPi and having the planned clinical contacts well above the ventral border to avoid the induction of phosphenes due to current spread to the optic tract. Nonetheless, because of the assumptions and limitations of the computational model, we cannot fully exclude potential contributions of activation of the internal capsule to the reductions in rigidity.

The results of this study provide some insight into the pathophysiological mechanisms of rigidity in PD. Imposed muscle stretch responses in the upper arm of people with PD are characterized by an initial short-latency (spinal) reflex that is normal in timing and magnitude, followed by an abnormally enhanced long-latency component (68–70). The magnitude of the long-latency response is stretch-velocity dependent, demonstrating that the response is largely mediated by the processing of group 1a (muscle spindle) afferent feedback (71, 72). The mechanisms contributing to the generation of the long-latency component of the muscle stretch response are still unclear, but the balance of evidence suggests that polysynaptic supraspinal pathways, including both a sensorimotor transcortical (73, 74) and a reticular formation-reticulospinal (75) circuit, are involved. Abnormally increased thalamo-motor cortical and/or reticular formation discharge in response to proprioceptive input is thought to generate the enhanced long-latency response in people with PD. Accordingly, activation of GABAergic pallidothalamic and pallido-reticular output pathways via GPi DBS may provide a mechanism to tonically suppress activity in these reflex loops (26). This mechanism is consistent with the idea that preferential driving of GPi output acts to suppress muscle activation, including resting (resting tone and tremor), stretch-evoked (rigidity), and levodopa-induced (dyskinesias) activity (15, 60).

The findings of this study need to be interpreted with caution given many of the assumptions and limitations of the subject-specific modeling approach. When considering axonal development in the computational model, it is important to note that the model was agnostic to axonal morphology, that is, axons in all pathways were modeled as single, nonbranching fibers without cell bodies and without the profuse collateralization of axons that is known to occur in the GPe and GPi (40). For this reason, the model likely underestimated the degree of pathway activation, especially in the GPe pathway. The omission of cell bodies, dendrites, and axonal afferents may only have had a minimal effect on model efficacy, since DBS is more likely to exert its effects on axons passing near the stimulating electrode than on local cell bodies (76). The model did not incorporate the topographic or somatotopic organization of pathways within the basal ganglia (63, 67, 77). For this reason, pathway activations that suppress rigidity in the forearm may be less effective for the leg or trunk. Additionally, our model did not incorporate somatotopic organization of motor subcircuits within the basal ganglia and GPi; this lack of detail could influence clinical outcomes and is thus missed in our analysis. The inherent assumptions and simplifications of the computational model may not fully capture the complex neural dynamics involved in DBS.

The findings of this study also need to be interpreted in the context of the study design and imaging approaches. First, there are often differences in the clinical programing approach to tune DBS systems given that each patient may have different preferences or goals in reduction of their symptoms. Although this likely increases variability in clinician-optimized DBS effects on rigidity across the cohort of participants, it also provides opportunities to compare pathway activations across multiple responder levels. Additionally, the pathway activation model results could have been affected by inaccuracies in lead localization or segmentation of the globus pallidus. To limit the likelihood of these issues, at least two investigators corroborated the alignment between the anatomical imaging and DBS lead localization data for each participant. Finally, the approach used ultrahigh-field imaging including 7-T MRI with diffusion tensor imaging (DTI) sequences, which may not be readily available at other centers and institutions.

The results of the dorsal versus ventral GPI DBS experiment were inconclusive, predominantly because of the use of relatively low stimulation intensities selected to contain the volume of tissue activated to the region of interest. Nonetheless, the distance of the contact from the ventral border did influence the relative activation of specific pathways, with dorsal DBS preferentially activating GPeSTNSN, PutGPe, and GPeGPiSTNSN pathways (Fig. 7).

One of the continuing challenges of trying to optimize GPi DBS motor outcomes is that not all motor features respond in the same manner to stimulation. GPi DBS delivered at “traditional” posterolateral targets (18, 19, 63), using standard isochronic stimulation parameters, has been shown to be very effective for the suppression of levodopa-induced dyskinesias, tremor, and rigidity (21, 78), but the effects on measures of akinesia, bradykinesia, gait, postural control, and other motor signs are often less pronounced and highly variable across individuals (79, 80). Therapeutic strategies using interleaved stimulation (combined GPe-GPi DBS) (62) or combined GPi- and STN-DBS (81) have been employed to try to solve this problem. The utility of these approaches needs to be informed by an increased understanding of the pathways mediating the therapeutic effects on separate motor signs. Subject-specific computational models, tailored to an individual’s neuroanatomy and DBS, have the potential to provide objective and unique solutions that improve the efficacy of outcomes and efficiency of programming.

## DATA AVAILABILITY

Data will be made available upon reasonable request.

## GRANTS

This work was supported by the following grants: NIH National Institute of Neurological Disorders and Stroke (NINDS) P50 NS123109, NIH NINDS P50 NS098573, NIH NINDS RO1 NS085188, NIH NINDS R01 NS124814, NIH F31 HD112-86-01A, NIH S10 OD025256, National Science Foundation (NSF) DGE-1734815, and Minnesota Discovery Research Innovation Economy (MnDrive).

## DISCLOSURES

No conflicts of interest, financial or otherwise, are declared by the authors.

## AUTHOR CONTRIBUTIONS

S.L.A., J.L.V., S.E.C., M.D.J., and C.D.M. conceived and designed research; M.E.L., S.L.A., T.P., R.P., J.W.C., S.E.C., N.H., and C.D.M. performed experiments; E.L., M.E.L., T.P., R.P., A.M.N., C.C.M., M.D.J., and C.D.M. analyzed data; E.L., M.E.L., S.L.A., J.L.V., M.D.J., and C.D.M. interpreted results of experiments; E.L., M.D.J., and C.D.M. prepared figures; E.L., M.E.L., M.D.J., and C.D.M. drafted manuscript; E.L., M.E.L., S.L.A., T.P., R.P., J.W.C., A.M.N., M.C.P., C.C.M., J.L.V., S.E.C., N.H., M.D.J., and C.D.M. edited and revised manuscript; E.L., M.C.P., M.D.J., and C.D.M. approved final version of manuscript.
